# An injectable, activated neutrophil-derived exosome mimetics/extracellular matrix hybrid hydrogel with antibacterial activity and wound healing promotion effect for diabetic wound therapy

**DOI:** 10.1186/s12951-023-02073-0

**Published:** 2023-08-30

**Authors:** Yanzhen Yu, Hangfei Jin, Linbin Li, Xin Zhang, Chunfang Zheng, Xi Gao, Yunxi Yang, Bingwei Sun

**Affiliations:** 1 Department of Burns and Plastic Surgery, The Affiliated Suzhou Hospital of Nanjing Medical University, Suzhou Municipal Hospital, Gusu School, Nanjing Medical University, 242 Guangji Road, Soochow, 215002 Jiangsu Province China; 2 Research Center for Neutrophil Engineering Technology, Affiliated Suzhou Hospital of Nanjing Medical University, 242 Guangji Rd, Soochow, 215002 Jiangsu Province China; 3 Gusu School, Nanjing Medical University, Soochow, China

**Keywords:** Activated neutrophils, Exosome, Extracellular matrix, Immune microenvironment, Chronic diabetic wound

## Abstract

Chronic diabetic wounds are primarily caused by infection, inflammation, and angiogenesis-related disorders. An ideal approach for treating chronic diabetic wounds is by combining anti-infection strategies, immune microenvironment regulation, and angiogenesis promotion. Vascular endothelial growth factor (VEGF) can promote the proliferation and migration of vascular endothelial cells, thereby promoting angiogenesis. However, the low stability and inability to target lesions limit its application. Polymorphonuclear neutrophil-derived exosomes (PMNExo) exhibit good delivery properties and can be used for the therapeutic delivery of VEGF. Furthermore, they retain the antibacterial ability of polymorphonuclear neutrophils (PMNs). Nonetheless, low PMNExo generation impedes its therapeutic applications. In this study, we prepared exosome mimetics (EM) from PMNs using the extrusion process; as a result, exosome yield significantly improved. To increase the residence of exosomes, an extracellular matrix (ECM) hydrogel, a thermosensitive material that can function as an in situ gel *in vivo*, was used as an exosome carrier. The active peptides in the ECM regulated the immune microenvironment of the wound. In summary, we loaded ECM with VEGF-encapsulated activated neutrophil exosome mimetics (aPMNEM) to develop VEGF–aPMNEM–ECM hybrid hydrogel for treating chronic wounds. The hydrogel accelerates the regeneration of chronic diabetic wounds. Our study provides a prospective therapy platform involving cytokines for treating different diseases.

**Graphical Abstract:**

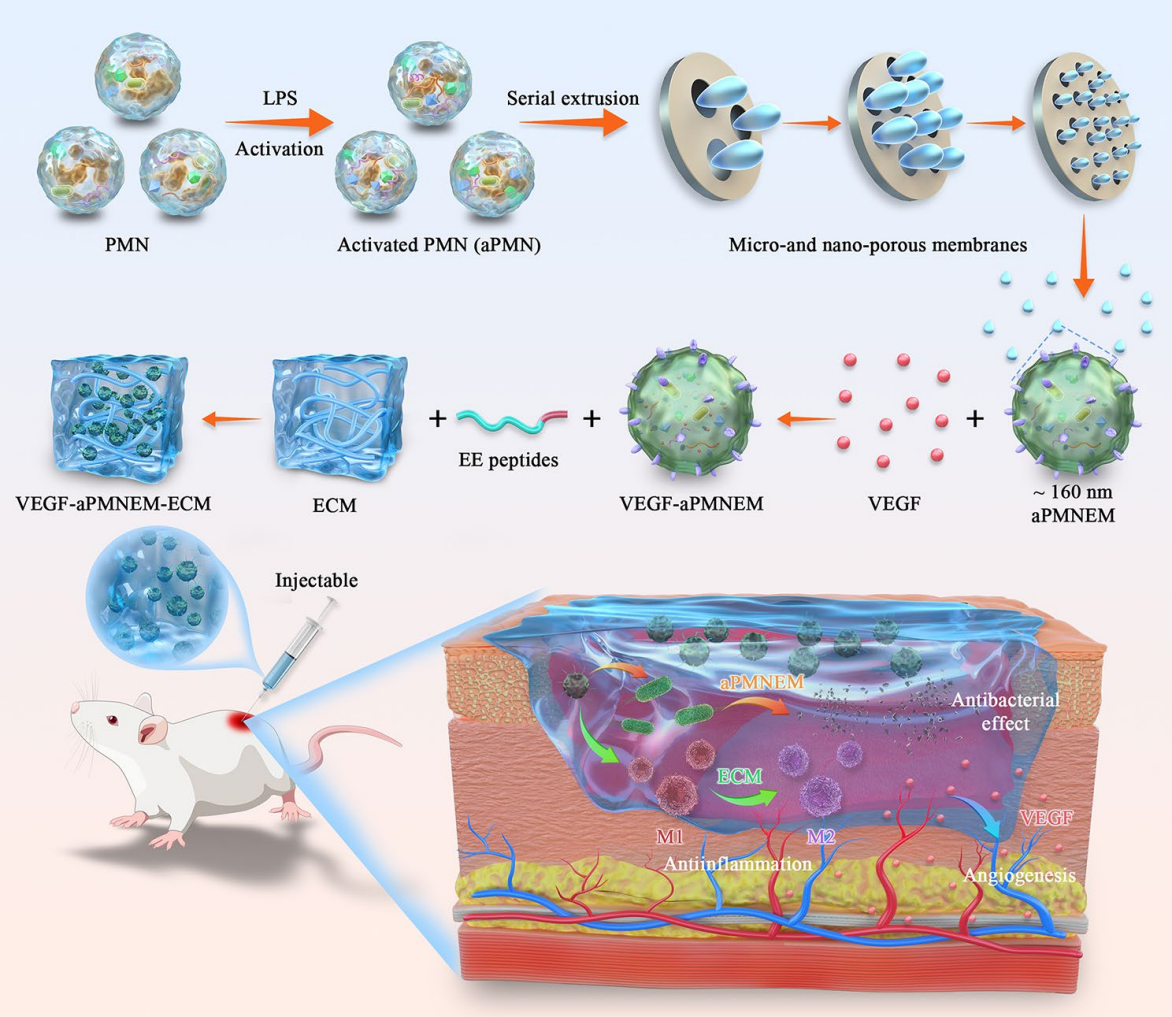

**Supplementary Information:**

The online version contains supplementary material available at 10.1186/s12951-023-02073-0.

## Introduction

Diabetes is a metabolic disease characterized by elevated blood glucose levels. Approximately 19–34% of individuals with diabetes will develop chronic diabetic wounds [1]. Although the precise etiology of poor wound healing remains unknown, persistent chronic wounds may primarily occur because of chronic inflammation within the wound microenvironment, resulting from oxidative stress, impeded angiogenesis, elevated proinflammatory cytokine expression, and microbial infection [2–5]. Therefore, the treatment of chronic diabetic wounds should be comprehensive from the perspectives of antibacterial action, regulating the immune microenvironment, and promoting angiogenesis.

There have been considerable advances in the integrated treatment of chronic wounds. Bioactive ingredients such as antibiotics and growth factors present a new approach to eliminating chronic inflammation and accelerating wound healing [6]. However, this approach has some limitations, including drug resistance, side effects, poor targeting, short half-life of drugs, and the inability to penetrate deep into the wound tissue [6]. Therefore, an antibiotic-free cytokine delivery system is warranted to efficiently and accurately deliver cytokines to the wound site, with controlled release while providing antibacterial action.

Polymorphonuclear neutrophils (PMNs) make up 40–70% of all white blood cells in humans and are the most prevalent type of granulocytes. Neutrophils are the primary line of defense for the host against invasive pathogens and possess an inherent capacity to phagocytose pathogens [7]. Studies have reported that PMN-derived exosomes (PMNExo) after pretreatment exhibit good antibacterial ability owing to the presence of components such as myeloperoxidase, elastase, dermcidin, and lysozyme [8, 9]. Cells secrete specialized vesicles, called exosomes, with a diameter of 50–200 nm, into the extracellular space. They mainly mediate intercellular communication. Exosomes display the advantages of low immunogenicity, biocompatibility, good biofilm penetration ability, and wound healing-promoting effects in skin injury treatment [10–13]. Taken together, PMNExo can not only exert their inherent antibacterial properties but also specifically treat diabetic chronic wounds after growth factor loading.

Previous studies have reported that vascular endothelial growth factor (VEGF) can stimulate angiogenesis via various signaling pathways, including the PI3K–AKT and MAPK–ERK pathways [14, 15]. The dynamic regulation of VEGF plays an important role in wound angiogenesis [16]. However, its application is limited because VEGF can be easily degraded, has low targeting abilities, and can be easily lost to diffusion *in vivo*. Nevertheless, exosome delivery improves drug stability and increases drug accumulation in the target cells, thereby improving drug efficacy and decreasing the required dose [17, 18]. In this case, exosome encapsulation will overcome the shortcomings of VEGF in treatment.

In addition, studies have reported that various proteins and polysaccharides in the extracellular matrix (ECM) construct their unique three-dimensional structure and surface topology. Apart from supporting the physiological morphology of tissues, the ECM may additionally recruit and regulate the adhesion, proliferation, and differentiation of cells [19]. After digestion, the ECM can be prepared into a thermosensitive material, i.e., an ECM hydrogel, that does not require complex crosslinking processes; this hydrogel has been widely used for chronic diseases such as osteoarthritis and intervertebral disc degeneration owing to its good biocompatibility and low cytotoxicity [20, 21]. Furthermore, the ECM hydrogel can be used as an in situ gel *in vivo* to maintain exosomes and avoid their loss along with physiological circulation and metabolism [22]. Moreover, bioactive oligopeptides generated by ECM breakdown can trigger the chemotaxis of different progenitor cells and macrophages during immunomodulation and angiogenesis, supporting M1 to M2 macrophage transformation and tissue regeneration [23, 24]. Therefore, the ECM hydrogel, therefore, functions as an elaborate regulatory system possessing therapeutic potential.

In the present study, we loaded the ECM with VEGF-encapsulated activated neutrophil exosome mimetics (aPMNEM) to develop VEGF–aPMNEM–ECM for treating chronic wounds. We noted that VEGF–aPMNEM–ECM can inhibit bacterial growth, promote vascular endothelial cell migration and tube formation *in vitro*, regulate macrophage polarization from M1 to M2 in the wound microenvironment, and effectively promote wound healing *in vivo*. The novel functional VEGF–aPMNEM–ECM hybrid hydrogel shows promise in treating chronic diabetic wounds. Furthermore, this platform that loads various functional cytokine-encapsulated exosomes into the ECM holds promise for successfully treating several diseases.

## Materials and methods

### Cell culture

PMNs were isolated from human peripheral blood using the EasySep™ direct human neutrophil isolation kit (Stemcell, Vancouver, Canada). PMNs were cultured in Roswell Park Memorial Institute (RPMI) 1640 medium (Gibco, Waltham, USA) supplemented with 10% exosome-free fetal bovine serum (FBS) (SBI, California, USA) and 1% penicillin and streptomycin (Beyotime, Shanghai, China) in a 37 °C incubator with a 5% CO_2_ air atmosphere. The lipopolysaccharide (LPS; Sigma, Saint Louis, USA) concentration of the activated PMNs (aPMNs) was 1 µg/mL for 30 min.

### Preparation of PMNExo, aPMNExo, PMNEM, and aPMNEM

The culture supernatants of PMNs and aPMNs were processed via multistage stepwise centrifugation to prepare and collect exosomes. Briefly, the cells were continuously cultured for 1 day in RPMI 1640 medium supplemented with 10% FBS and 1% penicillin/streptomycin in a 37 °C humidified incubator with 5% CO_2_. The cell culture supernatant was subsequently centrifuged for 10 min at 100 g, 10 min at 300 g, and 30 min at 10,000 g at 4 °C to ensure adequate removal of cell membrane fragments. Then, the supernatant was centrifuged at 100,000 g for 90 min at 4 °C to collect the exosomes PMNExo and aPMNExo. PMNs and aPMNs were placed into extruders containing 8 μm, 5 μm, 1 μm, 400 nm, and 200 nm filters to prepare PMNEM and aPMNEM, respectively. The extruding fluid was centrifuged for 30 min at 3000 g to remove cell membrane fragments. Thereafter, it was centrifuged at 100,000 g for 90 min at 4 °C to collect the exosomes. Western blotting, transmission electron microscopy (TEM), and nanoparticle tracking analysis (NTA) were performed to characterize PMNExo, aPMNExo, PMNEM, and aPMNEM. Furthermore, four-dimensional (4D) label-free quantitative proteomics analysis was performed to measure the protein content in aPMNEM and serviced by LC Sciences (Hangzhou, Zhejiang, China).

### Western blotting

PMNExo, aPMNExo, PMNEM, and aPMNEM were harvested and lysed with RIPA lysis buffer (Beyotime). The proteins in the supernatants were quantified using the Enhanced BCA Protein Assay Kit (Beyotime) after the lysates were centrifuged at 18,000 g for 30 min at 4 °C. First, proteins were separated into fractions on 10% sodium dodecyl sulfate (SDS)–polyacrylamide gel electrophoresis before being transferred onto polyvinylidene fluoride (PVDF) membranes. Then, 5% bovine serum albumin was used to block the PVDF membranes for 2 h. The PVDF membranes were incubated with the primary antibodies at 4 °C overnight. Thereafter, the membranes were incubated with the secondary antibodies at 25 °C for 2 h. The PVDF membranes were then photographed under the iBright FL1500 imaging system (Thermo Fisher, USA) and visualized via chemiluminescence as per the established procedures. Protein band densities were calculated using ImageJ software. The TSG101 antibody was purchased from Abcam (Cambridge, UK). The CD63 and HSP90 antibodies were purchased from Proteintech (Illinois, USA). Lastly, goat anti-mouse or goat anti-rabbit HRP-conjugated secondary antibodies were supplied by Beyotime.

### Preparation of VEGF–aPMNEM–ECM

The ultrasound method was used to encapsulate VEGF into aPMNEM to prepare VEGF–aPMNEM. Briefly, at room temperature, 50 µg of VEGF was mixed with 50 µg of aPMNEM in a total reaction volume of 10 mL for 30 min. The reaction system was then treated with ultrasound in a water bath ultrasonic apparatus (KQ-300DE, Suzhou, China). The ultrasonic settings were as follows: ultrasonic power of 120 W and three cycles of 15 s, with 2 min of cooling time between each cycle. Finally, incubation was performed at 37 °C for 1 h, and ultrafiltration was performed three times using 100 KD filters to remove excess amounts of free VEGF. The rat VEGF ELISA kit (EK383, Multi Sciences, Hangzhou, China) was used to detect the changes in VEGF content in aPMNEM to confirm that it was successfully encapsulated in aPMNEM.

Next, the temperature-sensitive ECM hydrogel was prepared by physical freezing combined with SDS–Triton elution. First, the muscle tissue of the left ventricle of a pig was selected; fat and blood vessels were removed and the tissue was sliced into small pieces and subjected to three repetitive freezing and thawing cycles at − 80°C. Then, a decellularized operation was conducted by immersing the muscle tissue into a 0.1% SDS solution. The solution was stirred for 24 h and again treated with 1% SDS for 48 h; the solution was changed every 24 h and then treated with 1% Triton X-100 for 1 h. Subsequently, the solution was washed with deionized water and lyophilized. Finally, the lyophilized ECM was ground into a powder and digested with pepsin at a ratio of 10:1 (w/w) in a pH 2 solution for 24–48 h until the ECM particles completely disappeared. The final concentration of the ECM was adjusted to 1% (w/v). The salt concentration of the solution was adjusted using 10× PBS to reach the physiological standard, and NaOH was used to adjust the pH to 7.3 to obtain the ECM hydrogel. 4D label-free quantitative proteomics analysis was performed to measure the protein content in the ECM and was serviced by Novogene (Beijing, China). The rheological properties of the ECM were assessed using a rheometer (Physica MCR 92, Anton Paar, Germany). The storage modulus (G’) and loss modulus (G”) of the ECM was analyzed, with changes in the times at 37 °C.

EE peptides (RRPKGRGKRRREKQRPTDCHLAHLHNRS, 20 µg), VEGF–aPMNEM (900 µg), and ECM (500 µL) were mixed at 4 °C for 8 h to prepare VEGF–aPMNEM–ECM. After freeze-drying ECM and VEGF–aPMNEM–ECM, scanning electron microscopy (SEM) was performed to characterize these materials. The *in vitro* release of VEGF–aPMNEM by ECM under the action of linker EE peptide was measured via NTA.

### TEM and SEM

The morphological features of PMNExo, aPMNExo, PMNEM, and aPMNEM were examined using TEM. First, 2.5% glutaraldehyde in PBS (pH 7.4) was used to fix PMNExo, aPMNExo, PMNEM, and aPMNEM overnight. Then, exosomes were refixed overnight at 4 °C in 1% osmium tetroxide. Thereafter, exosomes were dried in acetone at progressively higher quantities and then implanted in epoxy resin. Finally, extremely thin portions were created and adsorbed onto formvar-coated copper grids. Lead citrate and uranyl acetate were used to stain the exosomes, and images were captured under the Tecnai transmission electron microscope (FEI, USA).

The structural integrity of ECM and VEGF–aPMNEM–ECM were analyzed using SEM (Hitachi SU8010, Japan). Briefly, the samples were fixed overnight in 4% glutaraldehyde and dried before sputter coating. Conductivity analysis was performed with an accelerated voltage of 10 kV. The sample was magnified at 25,000×, and images were captured using a built-in camera.

### NTA and zeta analysis

PMNExo, aPMNExo, PMNEM, and aPMNEM were diluted with 1× PBS to attain the recommended measurement range (10^5^–10^7^ particles/mL) and subjected to zeta potential measurements (ZetaView instruments, Particle Metrix, USA). Particle sizes, concentration, and zeta potential were determined using NTA software (Particle Metrix, Meerbusch, Germany).

### *In vitro* and *in vivo* antibacterial activity of aPMNEM

The *in vitro* antibacterial potential of aPMNEM against *Escherichia coli* and *Staphylococcus aureus* was assessed using the spread plate method. Briefly, 1.0 mL of the bacterial suspension (10^7^ CFU/mL) was incubated with 20 mL of aPMNEM (40 µg/mL) for 3 h. Then, 200 µL of the bacterial solution was equally distributed on an LB agar plate and incubated at 37 °C for 16 h, followed by counting of bacterial colonies. SEM was performed to characterize *E. coli* and *S. aureus*. The *in vivo* antibacterial ability of aPMNEM against *E. coli* and *S. aureus* was assessed by measuring the colonies in infected wound. Briefly, the bacterial suspension (3 mL, 10^7^ CFU/mL) and aPMNEM (8 mg/kg of body weight) were injected into the wound of SD rats. After 3 days, the back tissues were collected for sampling, fixation, sectioning, and Giemsa staining.

### Migration and tube-formation assays

Cell migration was evaluated using the scratch technique. Briefly, human umbilical vein endothelial cells (HUVECs) were grown to 100% confluency after being plated in 6-well plates at a density of 3 × 10^5^ cells/well. Then, using a 200-µL pipette tip, three parallel scratches were made in each well. Thereafter, 500 µL of PBS, ECM, aPMNEM–ECM, or VEGF–aPMNEM–ECM were independently administered to each group. The cells were grown in a 37 °C incubator with a 5% CO_2_ air atmosphere in RPMI 1640 medium supplemented with 10% FBS and 1% penicillin/streptomycin. The width of the scratch was visualized with an optical microscope (Olympus, Japan) under brightfield and measured using ImageJ software.

The tube formation assay was used to assess capillary network formation in the Matrigel. Briefly, 6-well plates were precoated with 1% (m/v) Matrigel; then, they were seeded with HUVECs at a density of 2 × 10^5^ cells/well. Each group was treated with 500 µL of PBS, ECM, aPMNEM–ECM, or VEGF–aPMNEM–ECM separately. After 6 h, the total length and total branching length in five randomly chosen fields were quantified using ImageJ software.

### Development of the chronic diabetic wound model and VEGF–aPMNEM–ECM treatment

Briefly, 8-week-old SD rats were selected and fed for 7 days. For 4 weeks, all rats were given a high-fat and high-sugar diet. Thereafter, the rats were administered intraperitoneal injections of 2% streptozotocin (30 mg/kg) based on their body weight. When both fasting blood glucose and oral glucose tolerance test were determined to be > 11.1 mmol/L, the model of type II diabetes mellitus was considered to have been successfully established.

The effect of VEGF–aPMNEM–ECM on wound healing was assessed using a full-thickness skin wound rat model. Briefly, rats were anesthetized using an anesthesia machine for small animals and isoflurane. First, hair was removed from each rat’s back, and then a spherical, full-thickness wound area with a diameter of 2 cm and a thickness of 2.5 mm was prepared. After the rats were divided into four random groups, they received 500 µL of the treatment solutions via injections. The wound damage regions were determined using the Image J program after the wounds were examined and photographed at 1, 3, 5, 7, and 14 days. After 14 days, all rats were sacrificed. Skin specimens were removed and fixed with 4% paraformaldehyde for Masson, immunohistochemical (IHC), and hematoxylin–eosin (H&E) staining.

### Biodistribution study of VEGF-aPMNEM-ECM

VEGF–aPMNEM–ECM containing DiR-labeled exosomes was injected into the wound area. The images reflecting exosome biodistribution were captured using the IVIS imaging system (Lumina XRMS Series III, USA) at pre-established time points.

### Histological, masson, and IHC staining

Histological, Masson, and IHC staining were conducted according to previously reported methods [25, 26]. Skin sections were stained with H&E and Masson for histological examination. For IHC analysis of skin tissue sections, antibodies against α-SMA (CST, Beverly, MA, USA) and CD31 (Proteintech, USA) were used. The percentage positive area (%) of CD31 and α-SMA from IHC was assessed using ImageJ software. Furthermore, a microscope (Olympus) was used to visualize the stained sections and capture the images.

### Statistical analysis

All experiments were independently repeated three times, and the entire set of measurements was performed in triplicate. All data are represented as mean ± standard deviation (SD). Statistical significance was determined via one-way analysis of variance (ANOVA) using GraphPad Prism software (GraphPad Software 9, Inc., La Jolla, CA, USA). A P-value of < 0.05 was considered statistically significant.

## Results

**Fig. 1:**
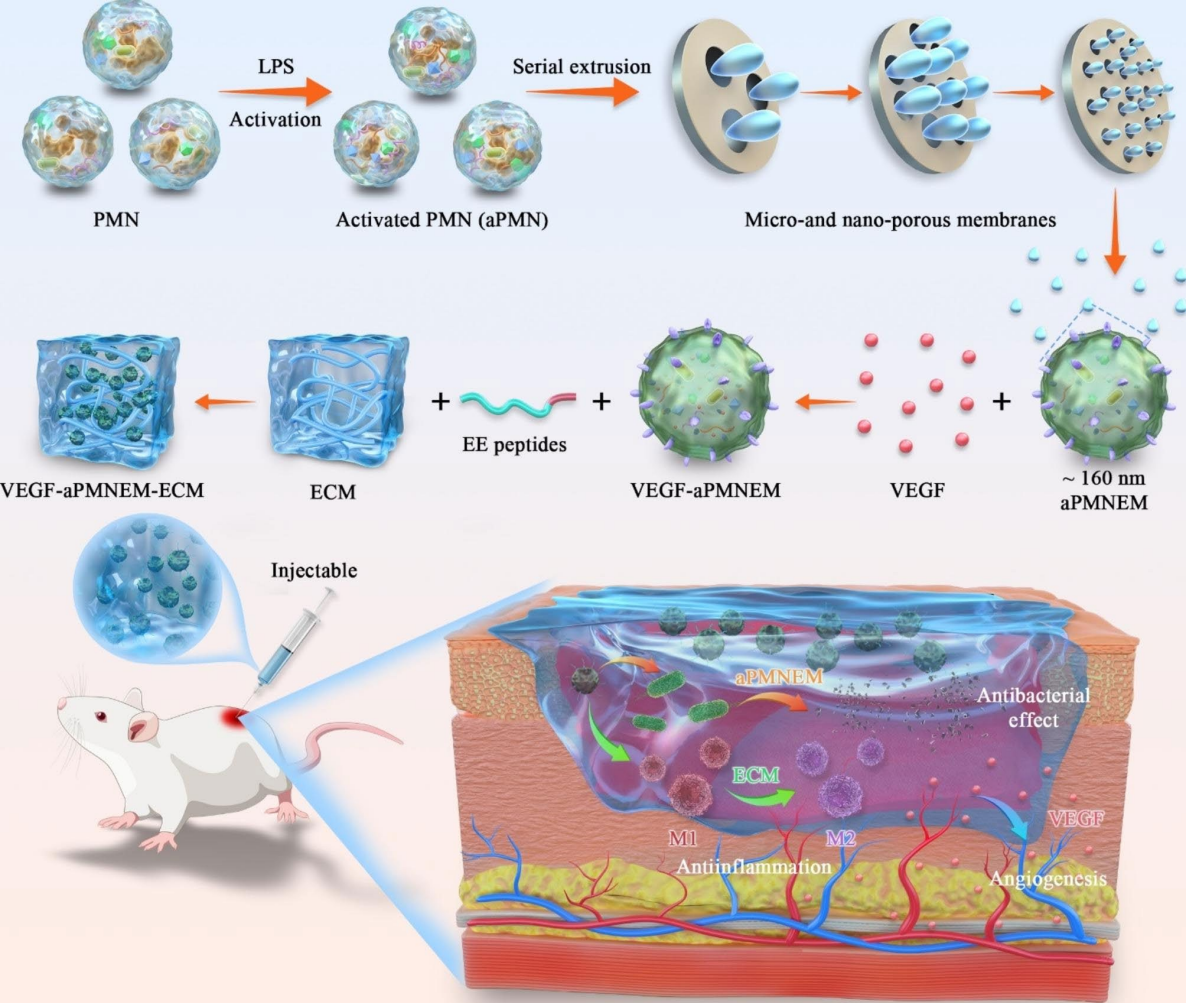
Scheme showing the study procedures. A total of four main steps were performed: (1) Lipopolysaccharide-activated polymorphonuclear neutrophils (LPS-aPMN) were passed through multilayer filtration membranes to prepare activated PMN exosome mimetics (aPMNEM); (2) Vascular endothelial growth factor wrapping into aPMNEM; (3) fabrication of VEGF–aPMNEM–ECM hybrid hydrogel; and (4) treatment of chronic diabetic wounds with VEGF–aPMNEM–ECM

We developed a multifunctional biomaterial comprising an ECM hydrogel and VEGF-loaded aPMNEM for the treatment of chronic diabetic wounds. The biomaterial has the following features: (1) for wound infection treatment, aPMNEM can play an antibacterial role via bactericidal-associated proteins; (2) aPMNEM, functioning as a carrier, can deliver VEGF and protect it from degradation, thereby promoting angiogenesis in chronic diabetic wounds; (3) ECM hydrogel, a thermosensitive material that can function as an in situ gel *in vivo* and increase the residence of aPMNEM. Figure 1 presents the scheme of the present study. First, we filtered LPS-activated PMNs through multilayer filtration membranes to prepare aPMNEM. Second, we used ultrasound to wrap VEGF into aPMNEM (VEGF–aPMNEM). Third, using EE peptides, the ECM and VEGF–aPMNEM were crosslinked to prepare VEGF-loaded PMN exosome mimetics-enriched ECM (VEGF–aPMNEM–ECM) hybrid hydrogel. Finally, VEGF–aPMNEM–ECM was developed and used for chronic diabetic wound treatment *in vivo*.

### Construction of VEGF–aPMNEM–ECM

**Fig. 2:**
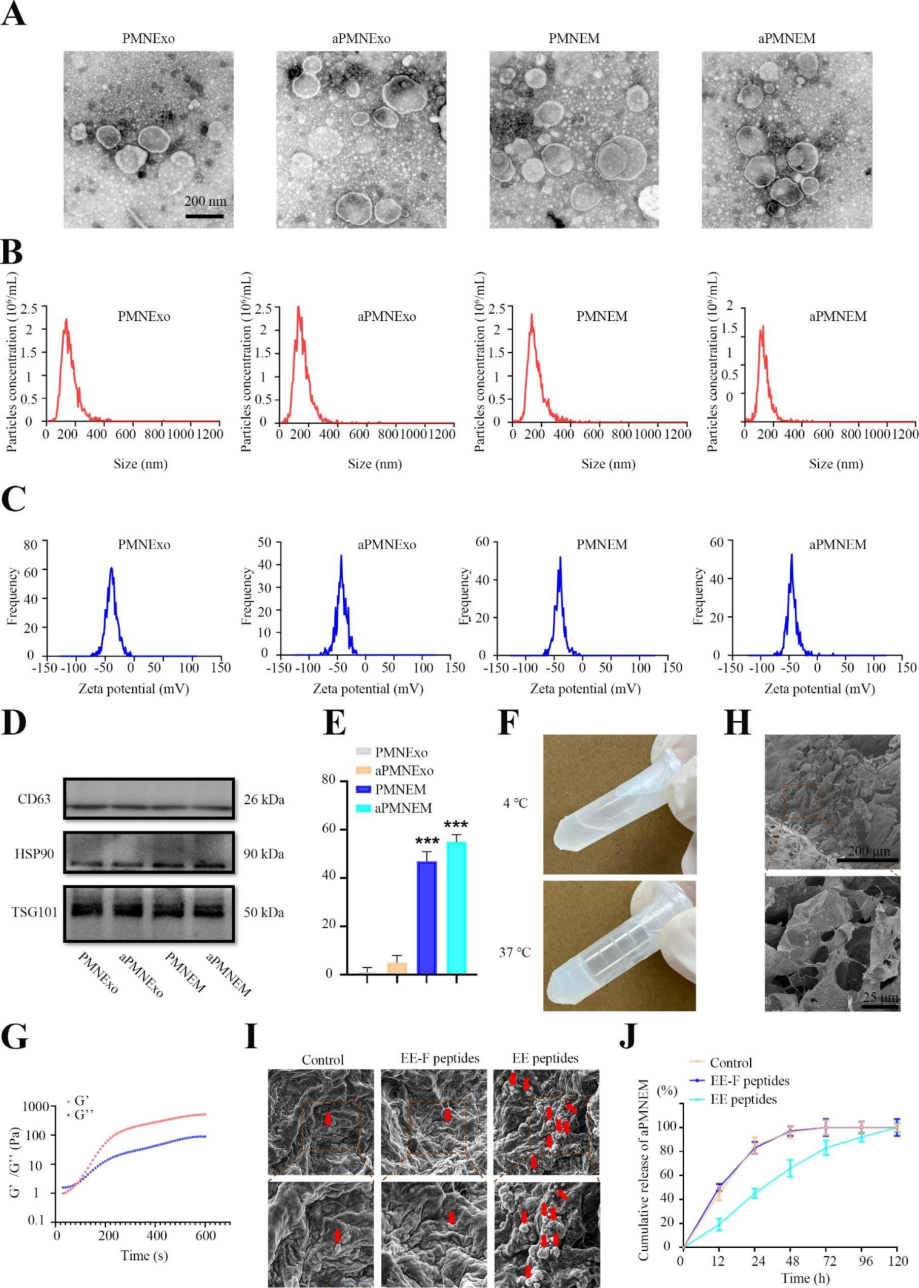
Fabrication and characterization of VEGF–aPMNEM–ECM. **(A)** Transmission electron microscopy images for PMNExo, aPMNExo, PMNEM, and aPMNEM morphology. **(B)** Nanoparticle tracking analysis of particle size and **(C)** Zeta potentials of PMNExo, aPMNExo, PMNEM, and aPMNEM. **(D)** Western blotting analysis for exosomal markers of PMNExo, aPMNExo, PMNEM, and aPMNEM. **(E)** The production of PMNExo, aPMNExo, PMNEM, and aPMNEM from the same number of PMN cells. **(F)** ECM images at 4 and 37 °C. **(G)** Elastic modulus of the ECM at 37 °C. **(H)** Scanning electron microscopy images of the ECM showing the overall structure of the ECM observed under low magnification conditions (top) and the local structure of the ECM observed under high magnification conditions (bottom). **(I)** Scanning electron microscopy images of the ECM-adsorbed aPMNEM. **(J)** The curve of the slow release of aPMNEM from the ECM. Data are represented as the mean ± standard deviation (n = 3). Analysis of variance was performed; “***” : p < 0.001

To verify the quality of aPMNEM, we characterized aPMNEM using TEM, western blotting, NTA, and zeta potential measurement. The aPMNEM had a size of approximately 160 nm diameter, which is similar to the sizes of PMNExo, aPMNExo, and PMNEM, based on TEM findings and NTA (Fig. 2A and B). PMNExo, aPMNExo, PMNEM, and aPMNEM exhibited circular vesicle morphologies that were comparable with those of typical exosomes previously reported [27, 28]. Furthermore, western blotting confirmed that PMNExo, aPMNExo, PMNEM, and aPMNEM had the same markers, such as CD63, HSP90, and TSG101 (Fig. 2D), CD63 is a tetraspanin protein enriched on the surface of exosomes that primarily participates in their production [29]. HSP90, in addition to its well-known protein chaperone effect, can also mediate membrane deformation and stimulate the release of exosomes [30]. TSG101 regulates the release of exosomes by regulating the degradation of lysosomes [31]. Zeta potential measurements revealed that aPMNEM had a potential of approximately − 40 mV, similar to the surface membrane potential of PMNExo, aPMNExo, and PMNEM (Fig. 2C). To quantify the productivity of aPMNEM, we measured the amount of PMNExo, aPMNExo, PMNEM, and aPMNEM produced from the same number of cells via NTA. NTA revealed that the output of aPMNEM produced via multilayer filtration extrusion was approximately 10 times higher than that of the production of aPMNExo (Fig. 2E).

Next, to investigate the quality of aPMNEM, we performed a 4D label-free quantitative proteomics analysis and compared the protein content in aPMNEM and PMNExo via principal component analysis (PCA), volcanic map analysis, and heatmap analysis. We used Gene Ontology (GO) annotation to understand the functional properties of different proteins. PCA revealed that the protein contents in aPMNEM and PMNExo were different (Fig. S1A). Furthermore, proteomics analysis identified that 1807 different protein types in aPMNEM had significantly different levels when compared with those in PMNExo. Compared with PMNExo, 1501 proteins were upregulated (fold change > 2) and 306 proteins were downregulated (fold change > 2) among the differentially expressed proteins in aPMNEM (Fig. S1B and C). Moreover, GO annotation analysis revealed that aPMNEM had similar biological processes, cellular components, and molecular functions with PMNExo (Fig. S1D and E). Among the bactericidal-associated proteins, neutrophil gelatinase-associated lipocalin (LCN2), lysozyme (LYZ), and peptidoglycan recognition protein (PGLYRP1) were upregulated, whereas complement C3 (C3), myeloperoxidase (MPO), and complement C4-B (C4B) were downregulated in PMNEM compared with PMNExo (Fig. S1F).

We prepared temperature-sensitive ECM hydrogels by physical freezing combined with SDS-Triton elution methods [32, 33]. We placed the ECM at 4°C and 37°C to verify its temperature-sensitive characteristics, and the results showed that the ECM was liquid at 4°C and solid at 37°C (Fig. 2F). We measured the elastic modulus of the ECM hydrogel at 37°C, and G’ exceeded G’’ at around 80 s, which indicated the cross-linking in the ECM hydrogel (Fig. 2G). Further, we scanned the ECM by SEM to observe its microstructure. The SEM images showed the porous structure of the ECM (Fig. 2H).

To verify that EE peptides (See Supplemental Fig. S2 for details of EE and EE-F peptides [same amino acid composition as that of the EE peptide but different sequences]) could achieve ECM adsorption and slow aPMNEM release, we first observed the ECM microstructure after aPMNEM adsorption. More aPMNEM (extracellular vesicles with 40–200 nm of diameter) adhered to the ECM pretreated with the EE peptide (Fig. 2I and Supplemental Fig. S3). Additionally, we measured aPMNEM release from the ECM over time. The results showed that without EE-peptide modification, ECM released aPMNEM in about 2 days, whereas after EE-peptide modification, ECM released aPMNEM for 5 days (Fig. 2J).

### Molecular debridement of aPMNEM

**Fig. 3:**
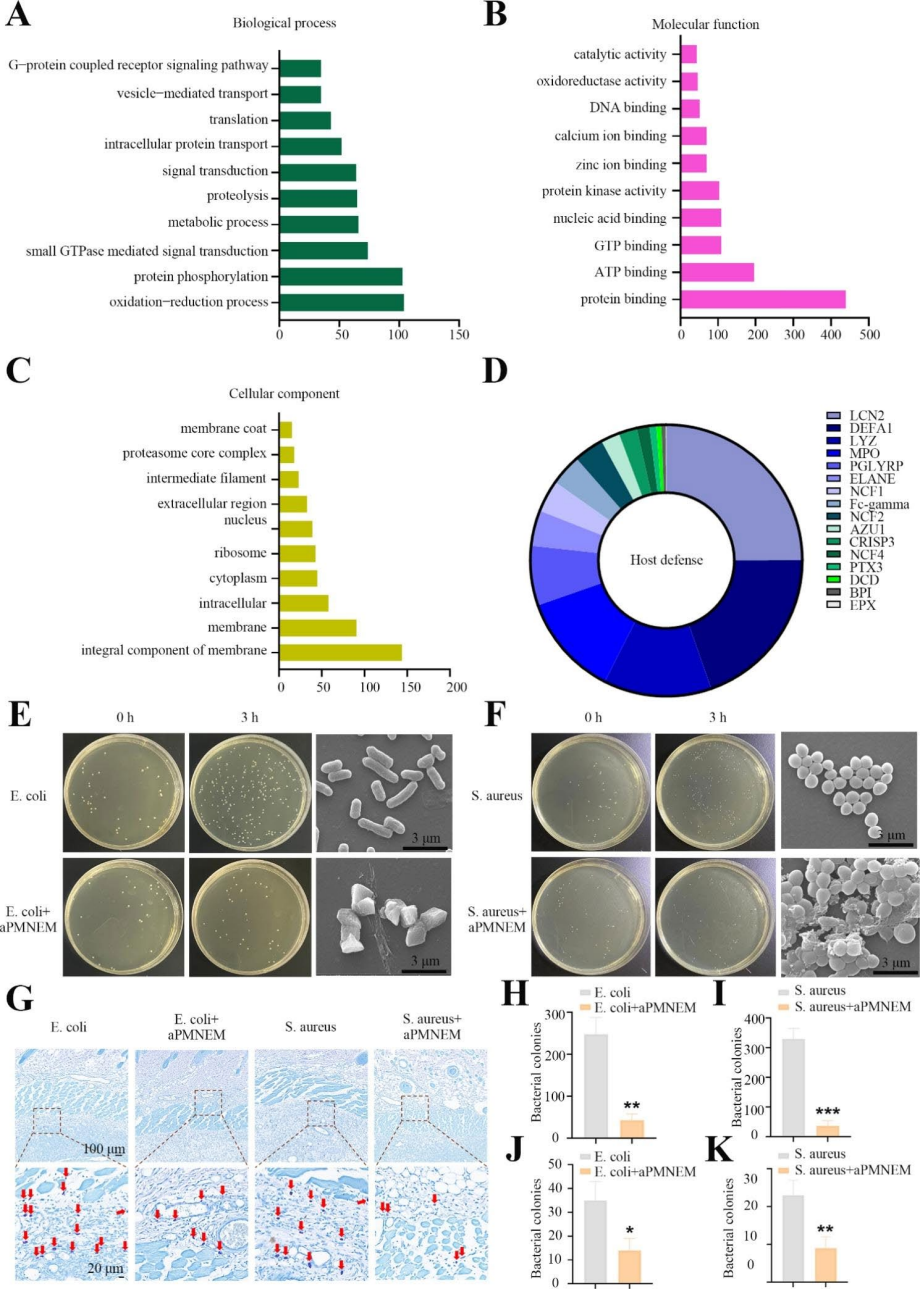
The bactericidal capacity of activated polymorphonuclear neutrophils-derived exosome mimetics (aPMNEM) *in vitro* and *in vivo*. Proteomic analysis of aPMNEM. Gene Ontology analysis of biological processes **(A)**, molecular functions **(B)**, and protein composition **(C)**. **(D)** aPMNEM contained many host defense proteins; in terms of content, lipocalin-2, defensin alpha 1, and lysozyme were identified as the top three. LCN2: lipocalin-2, DEFA1: defensin alpha 1, LYZ: lysozyme, MPO: myeloperoxidase, PGLYRP: peptidoglycan recognition protein 1, ELANE: neutrophil elastase, NCF1: neutrophil cytosol factor 1, NCF2: neutrophil cytosolic factor 2, AZU1: azurocidin, CRISP3: cysteine-rich secretory protein 3, NCF4: neutrophil cytosol factor 4, PTX3: pentraxin-related protein PTX3, DCD: dermcidin, BPI: bactericidal permeability-increasing protein, EPX: eosinophil peroxidase. aPMNEM inhibited the growth of *E*. *coli* and *S*. *aureus in vitro***(E, F, H, I)** and *in vivo ***(G, J, K)**. The red arrows represent the bacterial colonies formed within the infected wound tissue. Data are represented as the mean ± standard deviation (n = 3). T-test was performed; “*” : p < 0.05; “**” : p < 0.01; “***” : p < 0.001

We determined the composition of aPMNEM by analyzing its protein content by performing a 4D label-free quantitative proteomics analysis. The results revealed that it contained 3386 proteins, which participated in oxidoreductase activities as indicated by molecular function analysis, which probably contributed to the antibacterial activity of aPMNEM (Fig. 3A-C). The bactericidal-associated proteins, such as LCN2, neutrophil defensin 1 (DEFA1), LYZ, and MPO, contributed to host defense (Fig. 3D).

To verify the bacteriostatic ability of aPMNEM, we first explored the colony-growth inhibition of aPMNEM for *S. aureus* and *E. coli in vitro*. The findings indicated that aPMNEM treatment for three hours effectively impeded the growth of *E*. *coli* and *S*. *aureus in vitro*, destroying the bacterial cell wall structure (Fig. 3E, F, H, and I and Supplemental Fig. S4). Subsequently, aPMNEM was used to treat infected wound of rats with *E*. *coli* and *S*. *aureus*. The Giemsa results of the tissue showed that aPMNEM successfully inhibited bacterial growth *in vivo*, which corroborated well with the findings of *in vitro* bacteriostatic tests of aPMNEM (Fig. 3G, J, and K).

To assess the anti-inflammatory effect of aPMNEM, we determined changes in tumor necrosis factor-alpha (TNF-α), interleukin (IL)-1β, and IL-6 levels in infected wound and plasma of the control and aPMNEM groups at 24 and 48 h. The qPCR findings showed that the levels of inflammatory factors in infected wound of the control and aPMNEM groups did not change significantly at 24 h (Supplemental Fig. S5A). At 48 h, the levels of inflammatory factors in the aPMNEM group were considerably lower than those in the control group (Supplemental Fig. S5B). The plasma enzyme-linked immunosorbent assay and cytometric bead array (CBA) results showed an insignificant change in the levels of inflammatory factors in the control and aPMNEM groups at 24 and 48 h (Supplemental Fig. S5C-H).

### The effects and biological distribution of VEGF–aPMNM–ECM on vital organs *in vivo*

**Fig. 4:**
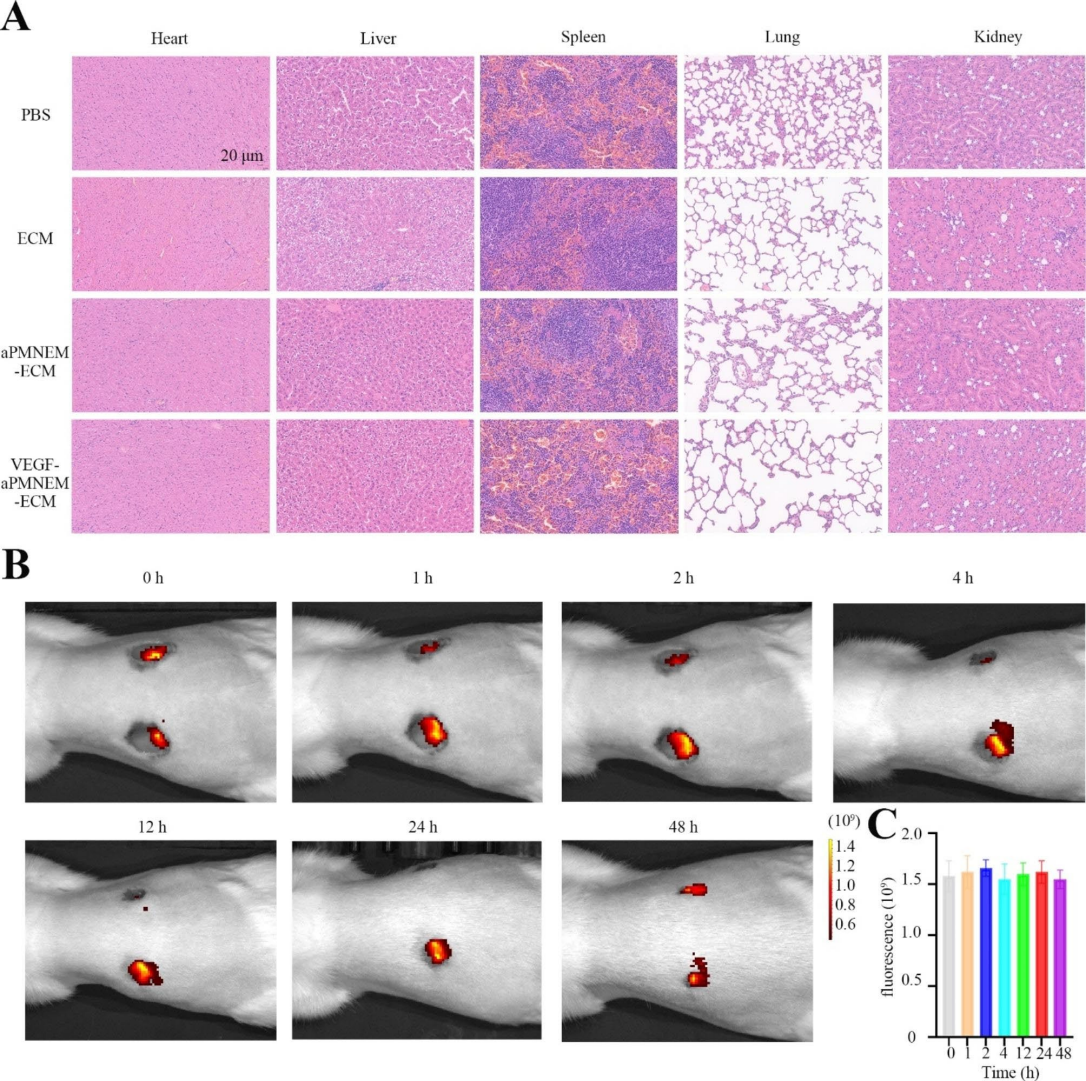
Systemic toxicity and biological distribution of VEGF–aPMNEM–ECM. **(A)** The biocompatibility of the VEGF–aPMNEM–ECM *in vivo*. *In vivo* images **(B)** and fluorescence **(C)** of VEGF–aPMNEM–ECM after wound injection

To verify the biocompatibility of VEGF–aPMNM–ECM, we collected different organs such as the heart, liver, spleen, lung, and kidney at 14 days post-treatment and observed the physiological structural changes in these organs via H&E staining assay. The results showed no apparent pathological changes in different tissues in treated rats compared with those in the PBS-treated controls (Fig. 4A), indicating that VEGF–aPMNM–ECM exhibited no toxicity for the full-thickness skin wound model of SD rats. To verify the biological distribution of VEGF–aPMNEM at the wound site, we injected VEGF–aPMNEM–ECM that contained DiR-labeled aPMNEM at the wound area and observed aPMNEM by small-animal *in vivo* imaging. The results showed that aPMNEM remained functional at the wound site for 48 h and did not spread over time (Fig. 4B and C).

### VEGF–aPMNEM–ECM promotes the healing of skin wounds in rats *in vitro* and *in vivo*

**Fig. 5:**
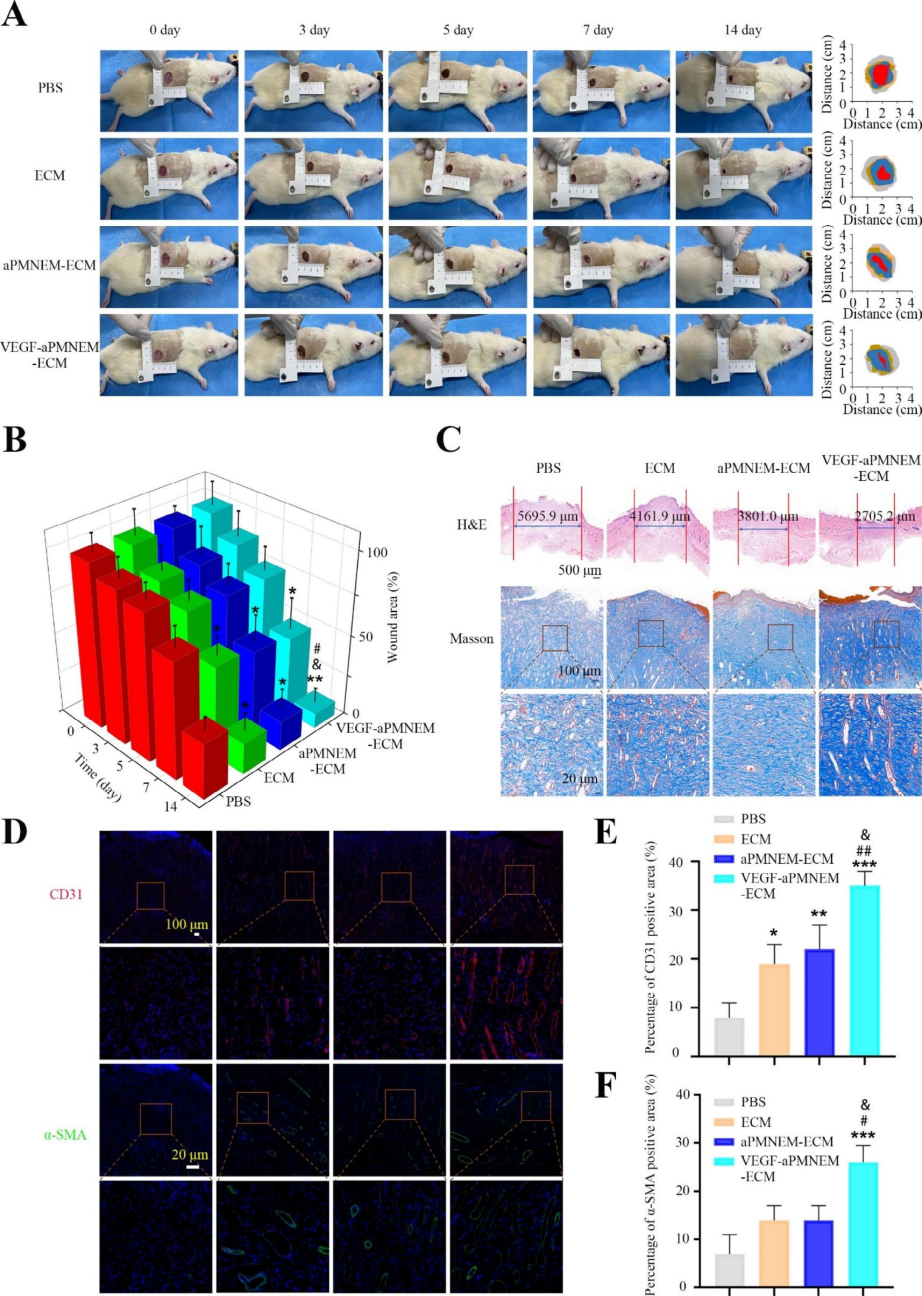
Effects of VEGF–aPMNEM–ECM on wound healing. **(A)** Digital images of wounds treated with PBS, ECM, aPMNEM–ECM, and VEGF–aPMNEM–ECM on days 1, 3, 5, 7, and 14. **(B)** Quantitative analysis of relative wound area rates on days 1, 3, 5, 7, and 14. **(C)** H&E staining and Masson staining of the wound section. The area marked by the red line in the middle reflected the wound closure. **(D)** VEGF–aPMNEM–ECM contributed to angiogenesis *in vivo*. Quantitative analysis of CD 31 **(E)** and α-SMA **(F)** positive area. Data are represented as the mean ± standard deviation (n = 5). Analysis of variance was performed; “*”, “#” and “&”: p < 0.05; “**”, “##” : p < 0.01; “***” : p < 0.001. “*” stands for comparison with the PBS group, “#” stands for comparison with the ECM group, “&” stands for comparison with the aPMNEM–ECM group

The diabetes SD rat model was established following published results to confirm that VEGF–aPMNEM–ECM may enhance the healing of skin wounds in rats by promoting angiogenesis [34]. Rats were deemed good diabetic models if their blood glucose levels were higher than 11.1 nmol/L. After that, we established an SD rat full-thickness skin wound model and administered several therapies to the rats. On days 0, 3, 5, 7, and 14 following the injury, their wound regions were measured and photographed (Fig. 5A and B). The results showed that compared with other therapies, VEGF–aPMNEM–ECM treatment might enhance wound healing more. The healing effects of the ECM and aPMNEM–ECM treatments were superior to those of the PBS control; however, these treatments were less effective than the VEGF–aPMNEM–ECM therapy.

The wound tissues were removed after 14 days of therapy and subjected to H&E and Masson staining, IHC analysis, and immunofluorescence (IF) analysis. The H&E staining results confirmed that VEGF–aPMNEM–ECM treatment showed the shortest wound edge and the highest degree of epithelialization in the four groups (Fig. 5C). The Masson staining results showed that compared with the other three groups, the VEGF–aPMNEM–ECM group exhibited the most collagen deposition at the wound site and the collagen was more orderly arranged (Fig. 5C).

The VEGF–aPMNEM–ECM treatment group showed the highest number of blood vessels, followed by the ECM and aPMNEM–ECM treatment groups, and there were a few blood vessels in the PBS treatment group, according to the results of IHC (Supplemental Fig. S6F) and IF (Fig. 5D-F) analyses by CD31 and an α-SMA antibody. These findings suggested that VEGF–aPMNEM–ECM enhanced cutaneous wound healing by promoting angiogenesis.

To verify the effect of VEGF–aPMNEM–ECM on cell migration, we treated HUVECs with VEGF–aPMNEM–ECM. The results showed that the ECM, aPMNEM–ECM, and VEGF–aPMNEM–ECM treatment groups significantly promoted HUVEC migration; however, no significant difference was observed between the three groups (Supplemental Fig. S6A and B). To confirm the therapeutic potential of VEGF–aPMNEM–ECM for endothelial cell angiogenesis, we performed tube-forming experiments using VEGF–aPMNEM–ECM. The results showed that VEGF–aPMNEM–ECM promoted the transformation of vascular endothelial cells to blood vessels, and the VEGF–aPMNEM–ECM group exhibited the most extended total and branched vessel lengths (Supplemental Fig. S6C-E).

### *In vivo* modulation effect of VEGF–aPMNEM–ECM on inflammation microenvironment

**Fig. 6:**
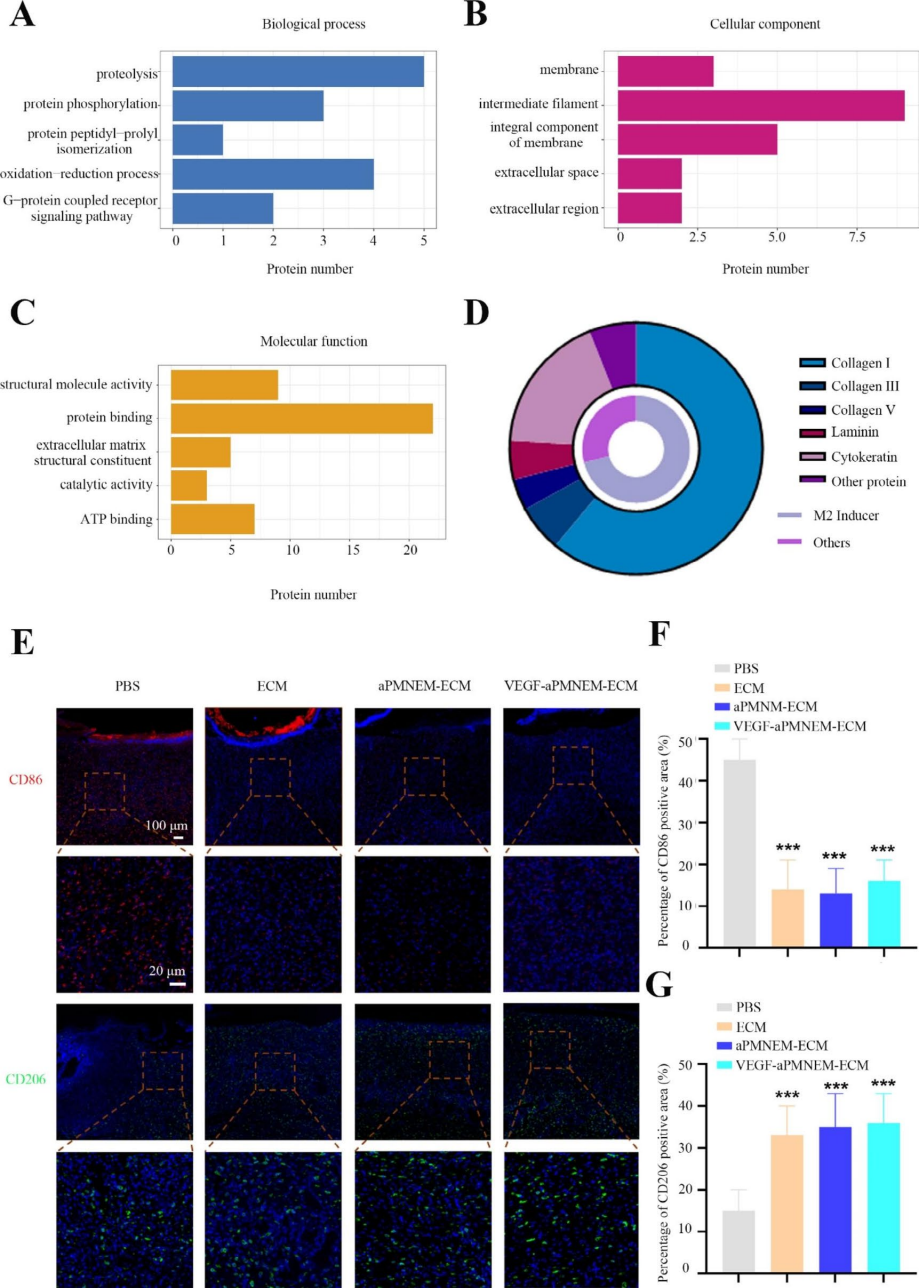
The VEGF–aPMNEM–ECM-induced capacity of macrophages from M1 to M2. Gene Ontology analysis of biological processes **(A)**, cellular components **(B)**, molecular functions **(C)**, and protein composition **(D)**. **(E)** VEGF–aPMNEM–ECM induced the transformation of macrophages from M1 to M2 *in vivo*. Quantitative analysis of CD 86 **(F)** and CD206 **(G)** positive area for immunofluorescence. Data are represented as the mean ± standard deviation (n = 5). Analysis of variance was performed; “***” : p < 0.001

To determine ECM composition, we analyzed ECM protein content by performing a 4D label-free quantitative proteomics analysis. A total of 112 proteins were found in the ECM hydrogel. Bioinformatics studies revealed a correlation between ECM and “extracellular constituent” (Fig. 6A–C). According to previous studies, those proteins with large quantities, such as collagen I, can caused the M1 macrophage transformation to M2 macrophages (Fig. 6D) [35].

The results of IF analysis by CD86 (M1 macrophage surface markers) and CD206 (M2 macrophage surface markers) antibodies confirmed that the ECM, aPMNEM–ECM, and VEGF–aPMNEM–ECM treatment groups significantly induced the M1 macrophage transformation to M2 macrophages; however, no significant difference was observed between the three groups (Fig. 6E-G).

## Discussion

Chronic diabetic wounds are characterized by inflammation, infection, and angiogenetic disorders. Anti-infection, immune microenvironment regulation, and angiogenesis promotion play a critical role in chronic diabetic wound treatment [1, 3, 4]. In this study, we successfully loaded ECM with VEGF-encapsulated activated neutrophil exosome mimetics to develop VEGF–aPMNEM–ECM and investigated its therapeutic effect on chronic diabetic wounds.

Generally, exosomes are obtained by the overspeed centrifugation of cell culture media, and exosome-free serum is used during cell culture. However, the amount of exosomes obtained is low, and the method is usually expensive. To facilitate exosome preparation and extraction, we used the extrusion method reported in the literature [36, 37] by extruding PMNs into a polycarbonate membrane filter, gradually reducing the pore size, and then using a 100-kDa filter to collect and purify aPMNEM. Exosomes separated by ultrafast centrifugation are often accompanied by many tiny fragments and other proteins, requiring further purification before practical application, which results in increased costs [33]. According to previous studies, exosome mimetics obtained by the extrusion method exhibit higher purity and concentration [36]. In the present study, PMNEM and PMNExo showed similar characteristics, both of which had membrane structures, particle sizes between 50 and 200 nm, and contained biochemical markers such as CD63, TSG101, and HSP90.

Bacterial infection may occur during the wound healing process, especially for chronic wound healing, resulting in delayed repair. During the treatment course, patients may face problems such as drug side effects, drug resistance, and financial burden due to the long-term use of costly antibiotics [38]. Thus, using an antibiotic-free molecular debridement strategy against wound infections has emerged as a promising model for eliminating bacterial infection and avoiding drug resistance [39]. PMNExo exhibit strong antibacterial properties. They can effectively kill *E*. *coli* (Gram-negative bacteria) and *S*. *aureus* (Gram-positive bacteria) with their bacteriostatic components [9]. In the present study, we showed the bacteriostatic activity of aPMNEM. The results of LB plate colony formation and infected wound animal experiments showed that aPMNEM exhibited a good bacteriostatic ability similar to that exhibited by PMNExo, which could significantly reduce the concentration of wound inflammatory factors. Subsequently, the aPMNEM and PMNExo mass spectrometry analysis showed that although proteins present in the aPMNEM and PMNExo changed, the biological processes involved were consistent and contained similar antibacterial activities.

VEGFs contribute to wound healing by promoting vascular endothelial cells to undergo proliferation and migration, thereby inducing angiogenesis. However, VEGF application has some disadvantages, such as its easy degradation, low targeting capacity, and easy diffusion loss *in vivo*. Although exosomes delivery improves drug stability and increases drug accumulation in target cells [17, 18], the resident time of exosomes is short, which hampers its effectiveness in wound healing [40].

The EE peptide is a 28-amino acid polypeptide, and its one side binds to transferrin receptors on the exosome membrane surface [41], and the other side attaches to collagen and laminin in the ECM [42]. The adsorption and slow release of aPMNEM from ECM were realized by EE-peptide modification, and finally, activated neutrophil-derived exosome mimetics/extracellular matrix hybrid hydrogel was prepared. The hydrogel prepared here is temperature-sensitive, liquid at 4 °C and solid at 37 °C, and portable and can be injected at the wound site to maintain aPMNEM and avoid their loss along with physiological circulation and metabolism.

Based on the abovementioned studies, we developed a novel VEGF–aPMNEM–ECM biomaterial for diabetic wound repair, which showed the following advantages: (1) VEGF was a potential regulatory factor in the treatment of diabetic trauma; (2) Exosome mimetics, as natural carriers, could prevent the enzymatic hydrolysis of VEGF, promote the long-distance reproduction of VEGF, and increase the action time of VEGF; (3) The ECM aided in tissue wound healing and exerted a beneficial effect on the disease microenvironment [43, 44]; and (4) The ECM could extend the action time of the exosome mimetics at the wound site.

Wound healing involves hemostasis, anti-inflammation, cell migration, cell proliferation, ECM deposition, and remodeling [45]. We evaluated the chronic wound healing effect of the VEGF–aPMNEM–ECM hybrid hydrogel in the present study. The H&E staining results were in agreement with the extent of wound healing in the digital images (Fig. 5A and B). The VEGF–aPMNEM–ECM group showed the most effective wound healing, followed by the ECM and aPMNEM–ECM groups, whereas the PBS group showed the worst wound healing effect. Notably, the VEGF–aPMNEM–ECM group showed the highest density of collagen deposition, indicating that VEGF–aPMNEM–ECM accelerated wound healing by promoting collagen deposition. Angiogenesis is critical for chronic wound repair because blood vessels provide nutrients to healing-related cells and maintain the growth of new granulation tissues [34]. In the present study, CD31 and α-SMA immunofluorescence staining was performed to visualize wound neovascularization. Fluorescence was rarely observed in the PBS group, and VEGF–aPMNEM–ECM-mediated CD31 expression was highest, indicating that exosome mimetics delivered VEGFs to the wound tissue and maintained the vascular function of VEGFs. The imaging results of small animals also showed that the ECM maintained exosome mimetics function at the wound site for a long time. These results suggest that applying VEGF–aPMNEM–ECM helps in diabetic wound healing by enhancing the healing process.

The inflammatory microenvironment plays a crucial role in tissue regeneration and wound healing. The protracted inflammatory phase of chronic wounds delays their transition into the proliferative phase [46]. Macrophages are crucial to the microenvironment of inflammation. TNF-α, IL-1β and IL-6 are three inflammatory cytokines that proinflammatory M1 macrophages might release to cause tissue damage and organ failure [47]. M2 macrophages secrete epidermal growth factors (EGF), VEGFs, and high amounts of anti-inflammatory cytokines to lower inflammation, control granulation development, and promote tissue regeneration [47]. To further verify the transformation effect of VEGF–aPMNEM–ECM on macrophages *in vivo*, CD86 and CD206 were stained with immunofluorescence. The results showed that most macrophages remained in the M1 phenotype (CD86^+^) when untreated, whereas a significant increase in M2-phenotype macrophages (CD206^+^) was observed in the collected tissues after VEGF–aPMNEM–ECM application. However, we found no significant difference in the red fluorescence signal of M2-phenotype macrophages detected in the ECM, aPMNEM–ECM, and VEGF–aPMNEM–ECM groups. Therefore, we believe that inducing an increase in M2-type macrophages in the traumatic microenvironment is primarily related to bioactive substances produced by ECM degradation in VEGF–aPMNEM–ECM, as previously reported [24, 47]. For instance, the C-terminal terminal peptide of type III collagen alpha enhances the in situ recruitment of perivascular progenitor cells *in vivo*, initiates osteogenic differentiation *in vitro*, and causes explicit chemotaxis of numerous cell types [24]. By affecting the cytoskeleton, the integrin α2β1 peptide (collagen 1(I)-CB3 fragment Asp-Gly-Glu-Ala) may induce macrophages to increase M2-type labeling [47].

The biosafety of VEGF–aPMNEM–ECM is critical for its biomedical applications. We evaluated the systemic toxicity of VEGF–aPMNEM–ECM *in vivo*. No significant changes in the behaviors of rats were observed during the treatment period. For the heart, lung, kidney, spleen, and liver, the H&E staining results showed that VEGF–aPMNEM–ECM did not cause any harm to major organs when applied to chronic diabetic wounds, indicating that in addition to effectively promoting wound healing, VEGF–aPMNEM–ECM exhibits good biocompatibility and biosafety.

Although VEGF–aPMNEM–ECM showed promising results in diabetic trauma treatment, a few problems required optimum solutions, such as multiple administration methods and dosing intervals to obtain the best therapeutic effect and the quantification of VEGFs in exosome mimetics to promote the consistency of administration. To increase the stability and efficacy of bactericidal-associated substances in PMNEM, a more suitable way of neutrophil activation should be explored. From the aspects of temperature, extrusion pressure, and speed, the technology of multi-layer filter membranes should be further improved to prepare better yielding exosome mimetics with useful clinical applications.

## Conclusions

In conclusion, we developed a functional biomaterial containing an ECM enriched with VEGF–aPMNEM for treating chronic diabetic wounds because an ECM provides a suitable microenvironment for aPMNEM to function in wound tissues, and aPMNEM are ideal carriers for cytokine delivery. The developed functional biomaterial offers a promising platform for cytokine therapy that can potentially be applied to treat different diseases by loading various available cytokines in aPMNEM–ECM.

### Electronic supplementary material

Below is the link to the electronic supplementary material.

Supplementary Material 1

## Data Availability

The data are available within the paper or are available from the authors upon request.
