# Supplementary material for: An injectable, activated neutrophil-derived exosome mimetics/extracellular matrix hybrid hydrogel with antibacterial activity and wound healing promotion effect for diabetic wound therapy
Source: J Nanobiotechnology. 2023 Aug 30;21:308. doi: 10.1186/s12951-023-02073-0 (PMC10466714; doi:10.1186/s12951-023-02073-0)
Supplement: Supplementary file 1 — Supplementary Material 1 [file 12951_2023_2073_MOESM1_ESM.docx]

**An injectable,** **activated neutrophil-derived exosome mimetics/extracellular matrix** **hybrid hydrogel with antibacterial activity and wound healing promotion effect for diabetic wound therapy**


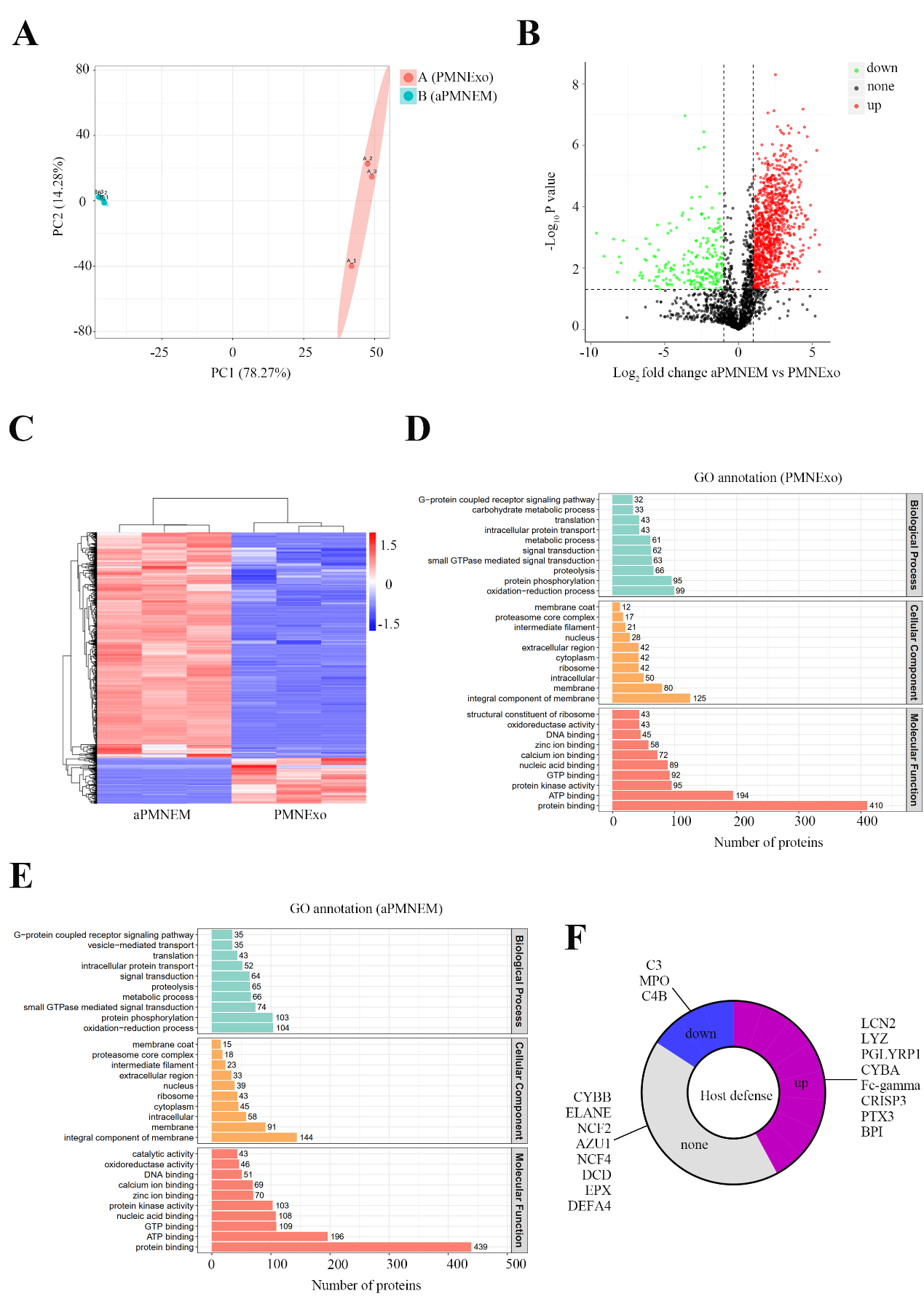


**Fig. S1** Proteomics analysis of polymorphonuclear neutrophils-derived exosomes (PMNExo) and activated polymorphonuclear neutrophils-derived exosomes mimetics (aPMNEM). **(A)** Principal component analysis and **(B)** volcanic map analysis of different proteins of PMNExo and aPMNEM. **(C)** Comparison of differential protein clustering between PMNExo and aPMNEM. **(D)** Gene Ontology (GO) functional annotation of PMNExo protein. **(E)** GO functional annotation of aPMNEM. **(F)** Comparison of host defense-related protein content between PMNExo and aPMNEM. LCN2: lipocalin-2, LYZ: lysozyme, PGLYRP1: peptidoglycan recognition protein 1, CYBA: cytochrome b-245 light chain, CRISP3: cysteine-rich secretory protein 3, PTX3: pentraxin-related protein PTX3, BPI: bactericidal permeability-increasing protein, C3: complement C3, MPO: myeloperoxidase , C4B: C4b-binding protein alpha chain, CYBB: Cytochrome b-245 heavy chain, ELANE: neutrophil elastase, NCF2: neutrophil cytosolic factor 2, AZU1: azurocidin, NCF4: neutrophil cytosol factor 4, DCD: dermcidin, EPX: eosinophil peroxidase, DEFA4: defensin alpha 4.


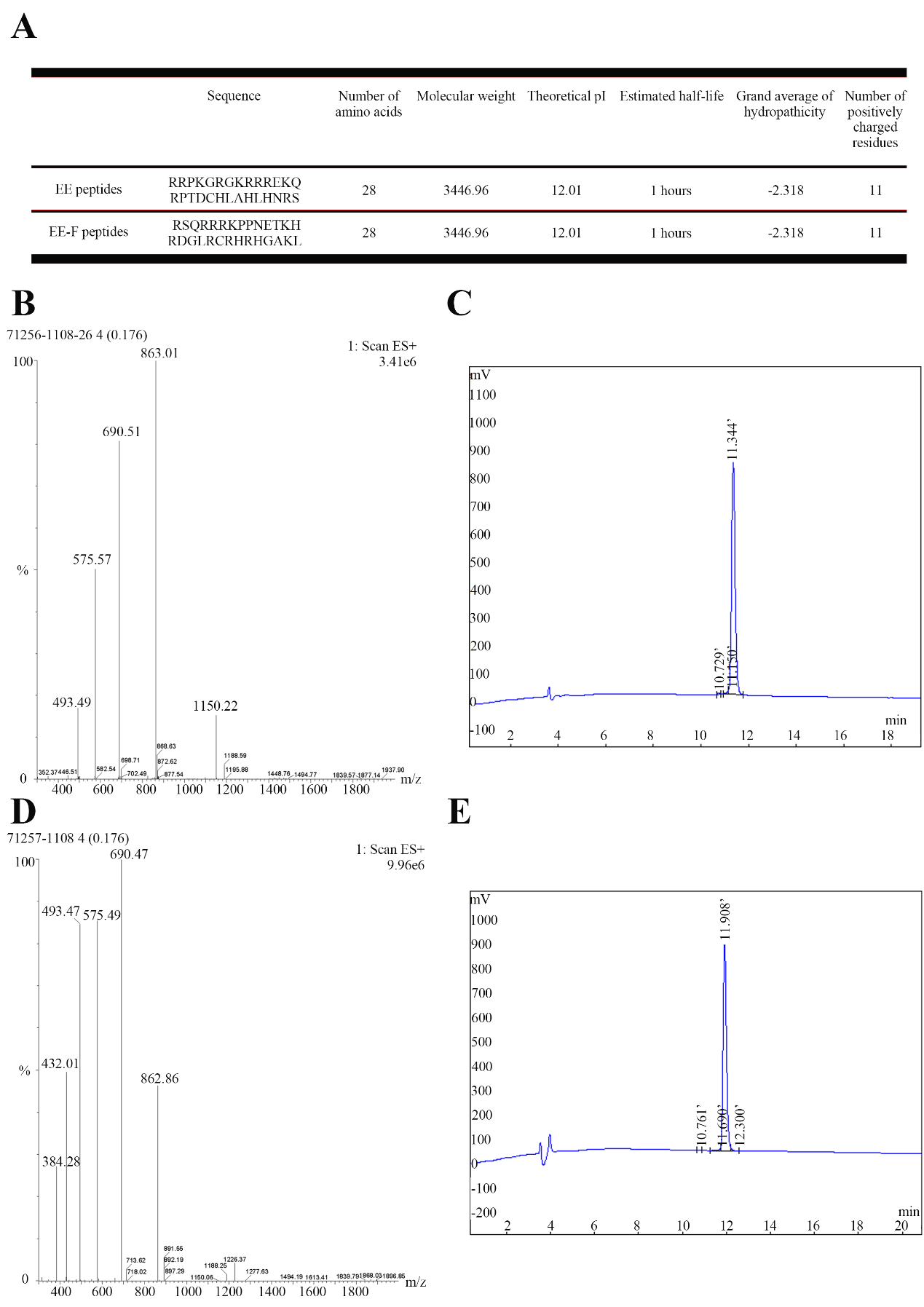


**Fig. S2** Synthesis and characterization of EE and EE-F peptides. **(A)** The parameters of EE and EE-F peptides. **(B)** Mass spectra of EE peptides. **(C)** High-performance liquid chromatographic (HPLC) analysis of EE peptides. **(D)** Mass spectra of EE-F peptides. **(E)** HPLC analysis of EE-F peptides.


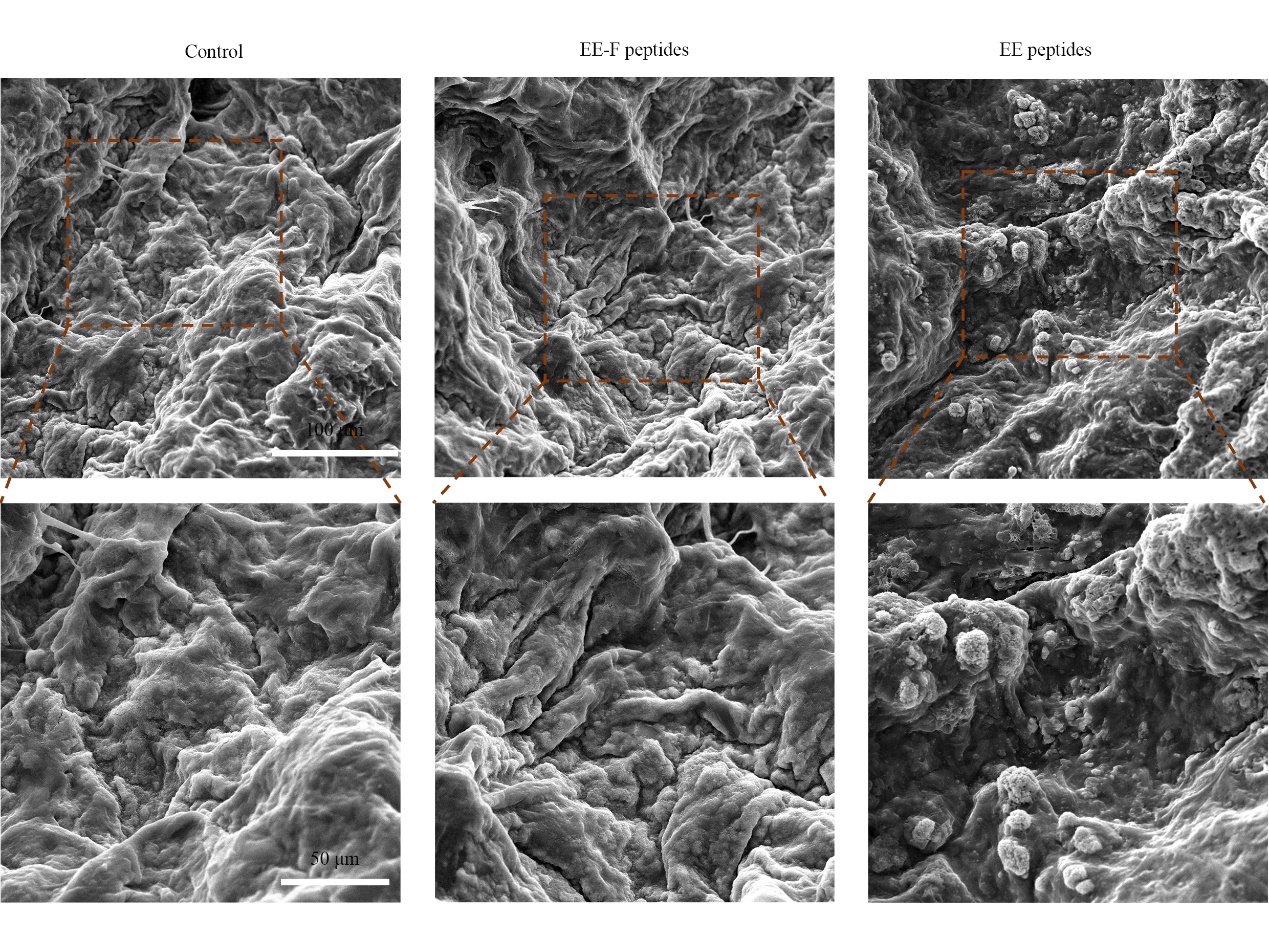


**Fig. S3** Scanning electron microscopy images of extracellular matrix-adsorbed activated polymorphonuclear neutrophils-derived exosome mimetics.


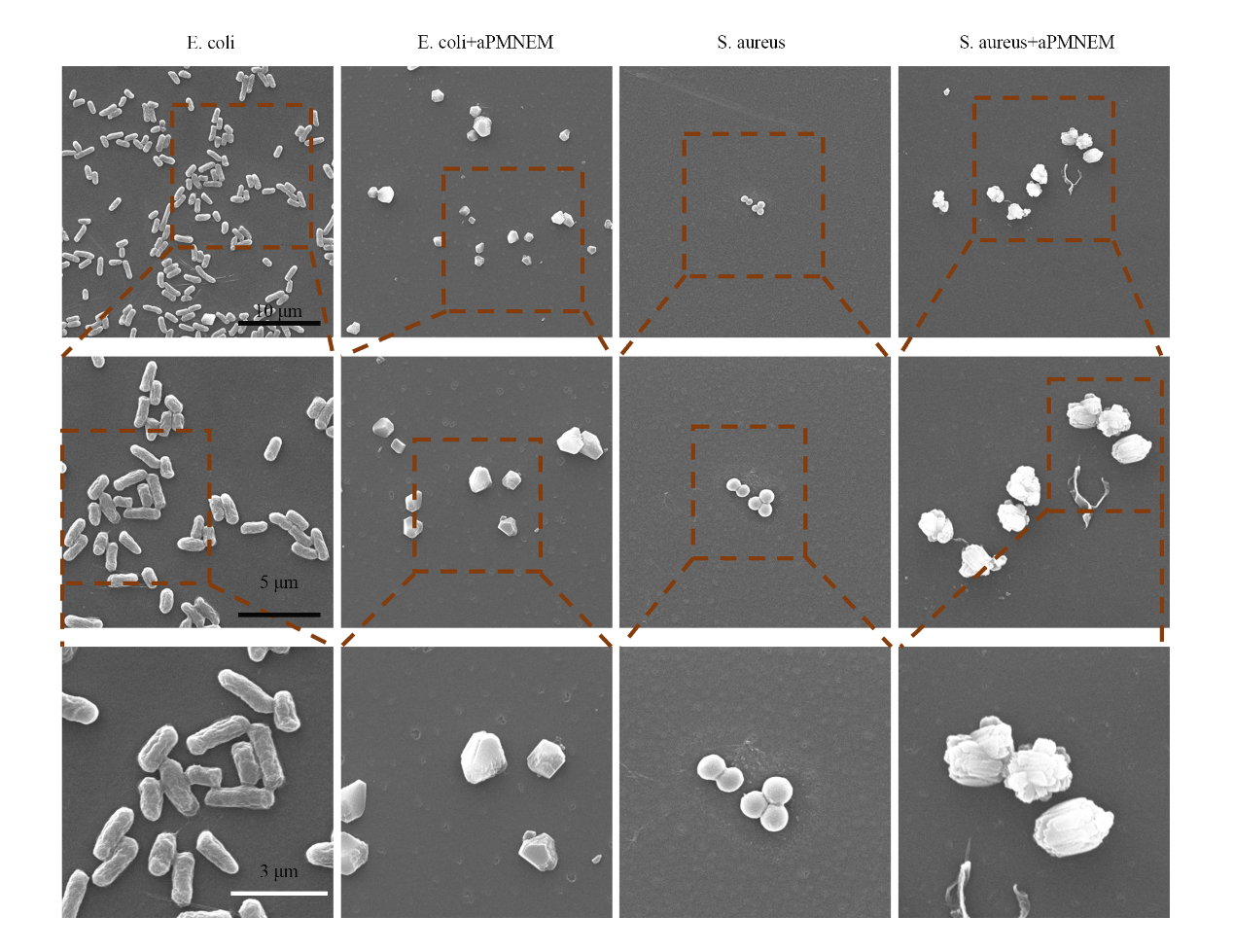


**Fig. S4** Scanning electron microscopy images of activated polymorphonuclear neutrophils-derived exosome mimetic-treated bacteria.


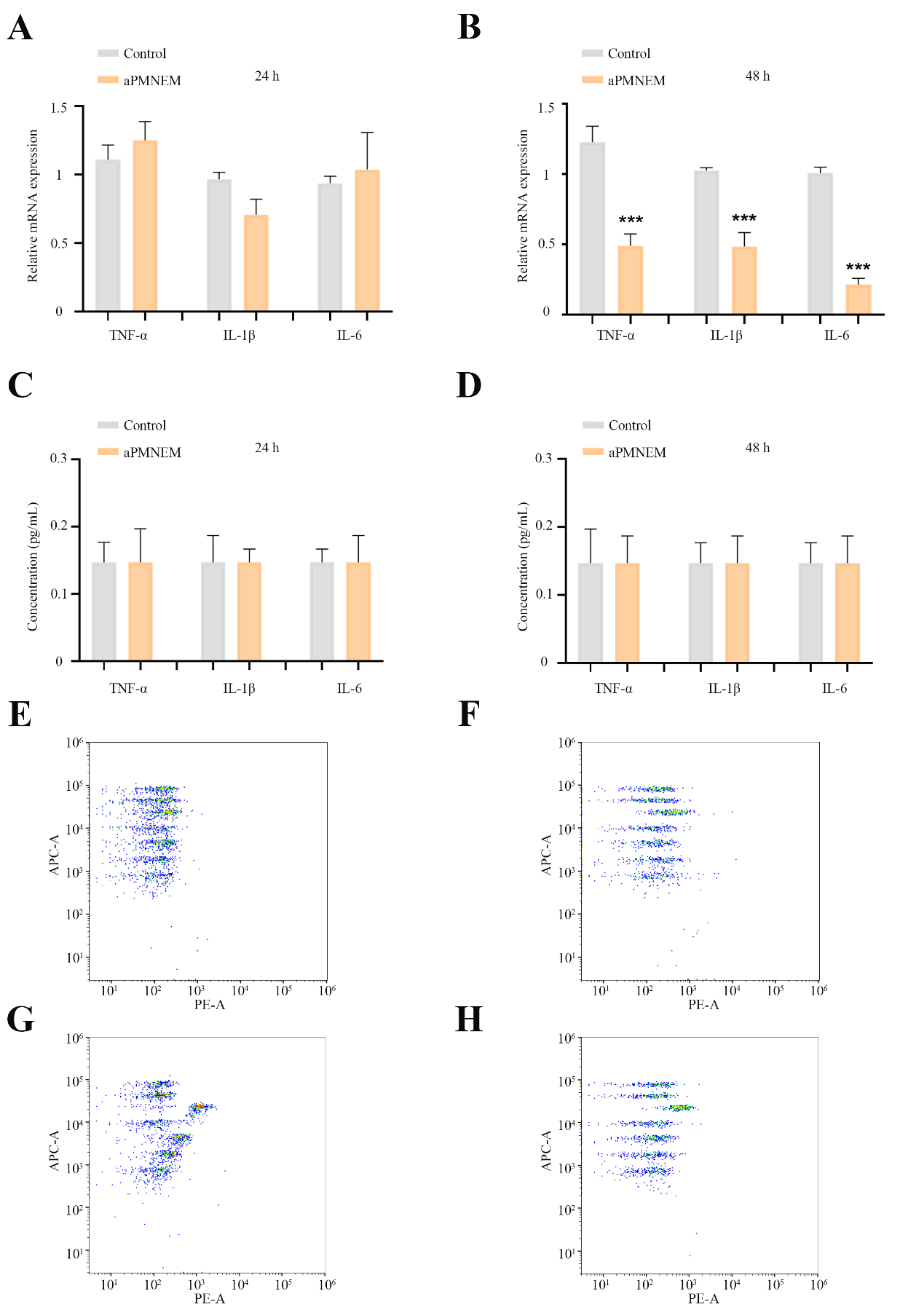


**Fig. S5** The changes of inflammatory factors in infected wound and plasma. Quantitative polymerase chain reaction determination of inflammatory factors in the infected wound at 24 h **(A)** and 48 h **(B)**. Enzyme-linked immunosorbent assay determination of inflammatory factors in plasma at 24 h **(C)** and 48 h **(D)**. The inflammatory cytokines cytometric bead array (CBA) in the plasma of the control group was determined at 24 h **(E)** and 48 h **(G)**. The inflammatory CBA in the plasma of the aPMNEM group was determined at 24 h **(F)** and 48 h **(H)**. Analysis of variance was performed; “***” : p < 0.001.


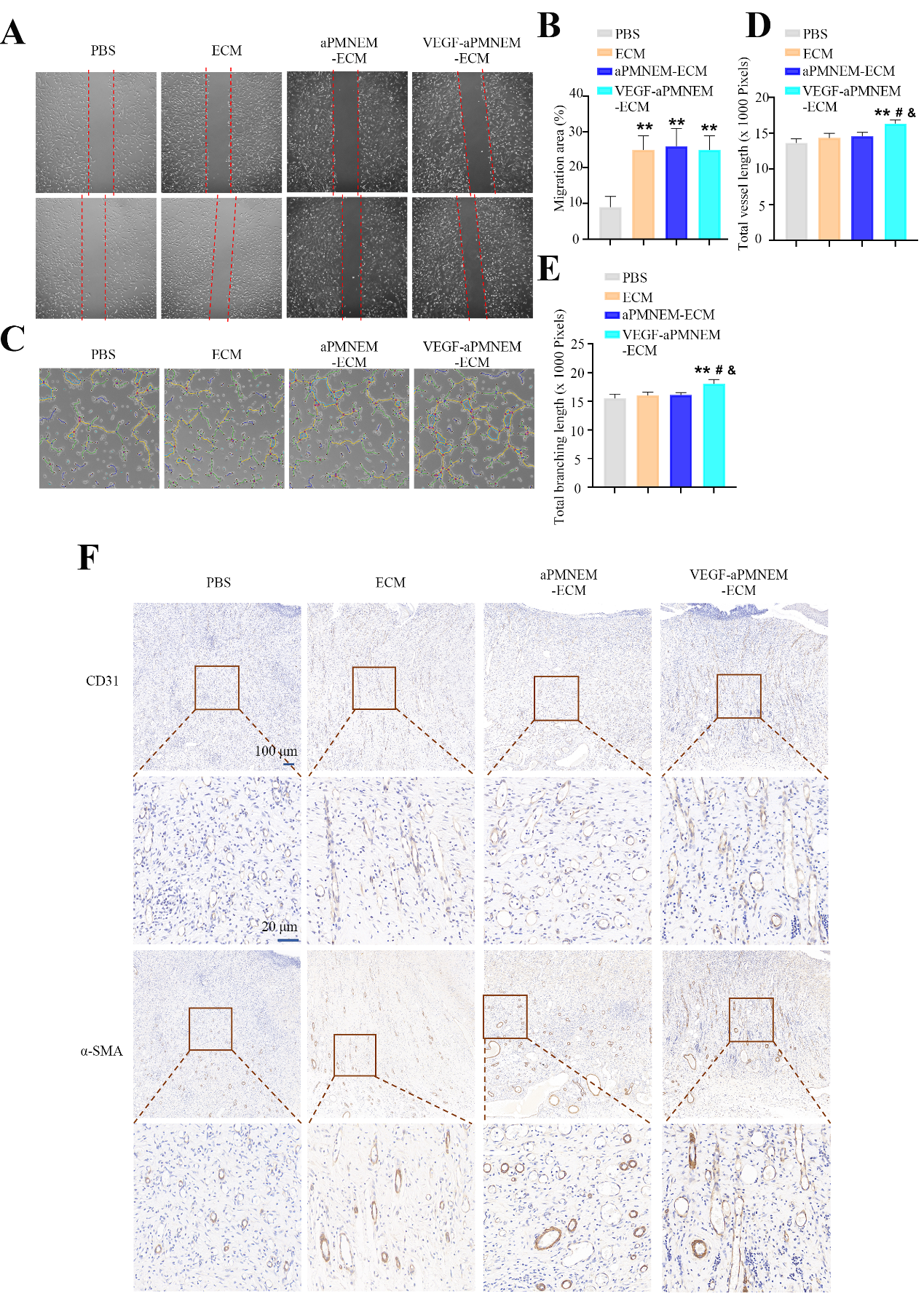


**Fig. S6** Effects of VEGF–aPMNEM–ECM on wound healing. Scratch wound **(A)** and tube-formation **(C)** assays. **(B)** Quantitative analysis of the migration rates. The statistics of total vessel length **(D)** and total branching length **(E)** in the tube forming experiment. **(F)** VEGF–aPMNEM–ECM contributed to angiogenesis *in vivo*. Data are represented as the mean ±standard deviation (n = 5). Analysis of variance was performed; “#” and “&”: p < 0.05; “**” : p < 0.01. “*” stands for comparison with the PBS group, “#” stands for comparison with the ECM group, “&” stands for comparison with the aPMNEM–ECM group.
